# Distinct Global Brain Dynamics and Spatiotemporal Organization of the Salience Network

**DOI:** 10.1371/journal.pbio.1002469

**Published:** 2016-06-07

**Authors:** Tianwen Chen, Weidong Cai, Srikanth Ryali, Kaustubh Supekar, Vinod Menon

**Affiliations:** 1 Department of Psychiatry & Behavioral Sciences, Stanford University School of Medicine, Stanford University, Stanford, California, United States of America; 2 Stanford Neurosciences Institute, Stanford University School of Medicine, Stanford University, Stanford, California, United States of America; 3 Department of Neurology & Neurological Sciences, Stanford University School of Medicine, Stanford University, Stanford, California, United States of America; Indiana University, UNITED STATES

## Abstract

One of the most fundamental features of the human brain is its ability to detect and attend to salient goal-relevant events in a flexible manner. The salience network (SN), anchored in the anterior insula and the dorsal anterior cingulate cortex, plays a crucial role in this process through rapid detection of goal-relevant events and facilitation of access to appropriate cognitive resources. Here, we leverage the subsecond resolution of large multisession fMRI datasets from the Human Connectome Project and apply novel graph-theoretical techniques to investigate the dynamic spatiotemporal organization of the SN. We show that the large-scale brain dynamics of the SN are characterized by several distinctive and robust properties. First, the SN demonstrated the highest levels of flexibility in time-varying connectivity with other brain networks, including the frontoparietal network (FPN), the cingulate–opercular network (CON), and the ventral and dorsal attention networks (VAN and DAN). Second, dynamic functional interactions of the SN were among the most spatially varied in the brain. Third, SN nodes maintained a consistently high level of network centrality over time, indicating that this network is a hub for facilitating flexible cross-network interactions. Fourth, time-varying connectivity profiles of the SN were distinct from all other prefrontal control systems. Fifth, temporal flexibility of the SN uniquely predicted individual differences in cognitive flexibility. Importantly, each of these results was also observed in a second retest dataset, demonstrating the robustness of our findings. Our study provides fundamental new insights into the distinct dynamic functional architecture of the SN and demonstrates how this network is uniquely positioned to facilitate interactions with multiple functional systems and thereby support a wide range of cognitive processes in the human brain.

## Abstract 2

Human cognitive versatility is supported by a distinct, highly flexible, yet stable, dynamic brain organization of the salience network and its interactions with multiple other functional systems.

## Author Summary

One of the most distinguishing features of the human brain is its ability to detect and attend to salient events in the environment. The salience network—a core large-scale brain network anchored in the anterior insula and the dorsal anterior cingulate cortex—is thought to play a crucial role in this process. To gain insights into the mechanisms that support this complex set of functions carried out by the salience network, we analyzed state-of-the-art fMRI data collected during multiple sessions with subsecond resolution and mapped dynamic time-varying functional interactions among the key neural components within the salience network and between the salience network and other core brain networks. We show that the large-scale brain dynamics of the salience network is characterized by several distinctive, behaviorally relevant, and robust properties, highlighting its highly flexible yet stable organization. Our findings provide fundamental new insights into the dynamic functional architecture of the salience network and demonstrate how it is uniquely positioned to facilitate interactions with multiple functional systems and thereby support cognitive flexibility.

## Introduction

The human brain is a complex system capable of supporting a wide range of adaptive goal-relevant behaviors. These behaviors are thought to be supported by the intrinsic functional architecture of large-scale functional systems that constrain and support diverse cognitive processes in a stable, yet flexible, manner [1–3]. The salience network (SN), in particular, plays a crucial role in cognition and emotion via detection and attentional capture of goal-relevant stimuli and facilitation of access to appropriate cognitive resources across a wide range of cognitive tasks [4–10]. The anterior insula (AI) and dorsal anterior cingulate cortex (dACC) nodes of the SN are among the most commonly activated regions in human neuroimaging studies [5,11,12], pointing to the ubiquitous involvement of this network in cognition. Emerging evidence also suggests that atypical functional engagement of the SN is a common feature of several neuropsychiatric disorders [13–16]. Identification of the dynamic spatiotemporal properties of the SN is therefore an important open question in systems and clinical human neuroscience.

The complex repertoire of functions subserved by the SN is thought to be realized through its time-varying functional interactions with other core intrinsic functional networks [17]. Functional neuroimaging studies to date have, however, focused on the static organization of the SN and other brain networks in an oversimplified time-averaged manner, partly due to the limited temporal resolution of fMRI and partly due to the lack of computational methods for mapping large-scale dynamics and difficulties in relating them to the known functional architecture of the human brain [18–20]. Here, we leverage high temporal-resolution fMRI data obtained from the Human Connectome Project (HCP) combined with novel dynamic graph-theoretical techniques to investigate dynamic interactions of the SN at subsecond temporal resolution. We focus on the spatial and topological properties of SN interactions within a whole-brain system that includes key nodes of the SN as well as a large set of brain regions that have been implicated in a wide range of cognition paradigms [3].

The SN is a large-scale paralimbic–limbic network anchored in the AI and dACC [8,17,21,22]. The SN is most readily identified using intrinsic functional connectivity analysis of fMRI data [8,21] and has an architecture that is distinct from other cognitive control systems including the frontoparietal network (FPN) and the ventral and dorsal attention networks (VAN and DAN). Intrinsic functional connectivity analyses using multiple methodologies have provided converging evidence that the dorsal AI has particularly robust connectivity with the dACC node of the SN [5,8,21]. Furthermore, task-based meta-analytical studies have consistently reported that the AI and dACC are among the most frequently coactivated regions across a wide range of human fMRI studies spanning multiple cognitive domains [5,11,12,23].

While static network analysis has isolated the SN and its core AI–dACC link as a system that is functionally distinct from other brain systems, emerging evidence suggests that the SN participates in a wide range of cognitive and affective tasks by facilitating access to attention and working memory resources once a salient event is detected [4,6,7,9,10]. Chronometric and dynamic causal analyses suggest that the SN facilitates task-relevant information processing by initiating appropriate transient control signals that engage cognitive and task control systems while suppressing the default mode network (DMN) [4,6,9,10,22,24]. In particular, the SN has been shown to play a crucial role in switching between large-scale brain networks involved in externally-oriented attention and internally-oriented mental processes [9,17]. The SN, together with the lateral FPN, typically shows increases in activation, whereas the DMN consistently shows decreases in activation below resting baseline during the performance of a wide range of cognitively demanding tasks [5,11,25–27]. Furthermore, brain responses within these regions increase and decrease proportionately, and often antagonistically, in relation to specific cognitive demands and subjective task difficulty [5,28–31]. More direct evidence for the role of the SN in such cross-network functional integration comes from studies of patients with traumatic brain injury [32]. Bonnelle and colleagues [32] found that the degree of white matter damage in the SN tract connecting the right AI to the dACC specifically predicted abnormal DMN function. Taken together, these findings provide converging evidence that the SN contributes to a variety of complex brain functions through interactions among its core nodes and with other brain networks.

Crucially, however, the temporal dynamics of SN connectivity and its interactions with other brain areas are not known, and it is not clear whether the SN has unique features with respect to its ability to flexibly interact with a diverse set of brain areas. This gap in knowledge is in part because most functional connectivity studies have examined brain network organization in a static, time-averaged manner under the assumption that functional interactions are stationary over time [20,33–36]. Analysis of time-varying functional connectivity has the potential to provide novel insights into brain dynamics [20,33,35,37–40], but little is known about the spatiotemporal organization of the SN, and other cognitive control systems, and their links to flexible cognitive behaviors. Importantly, precise quantitative characterization of the dynamic functional properties of the SN remains an important and unaddressed open question, with significant implications for human systems neuroscience and for our understanding of the many psychopathologies that have now been shown to be impacted by disruptions to this network [13,15,16,41–43]. Here, we use a large multisession dataset from the HCP [44] and apply novel quantitative techniques to systematically investigate large-scale brain dynamics in the context of a large set of cortical and subcortical nodes that have been implicated in a wide range of cognitive paradigms [25,45]. In addition to the SN, we include key nodes of other large-scale networks implicated in cognitive control and attention—FPN, DAN, VAN, and cingulate–opercular network (CON)—as well as DMN, a system important for self-referential mental processes and multiple sensorimotor networks [3,45].

**Fig 1:**
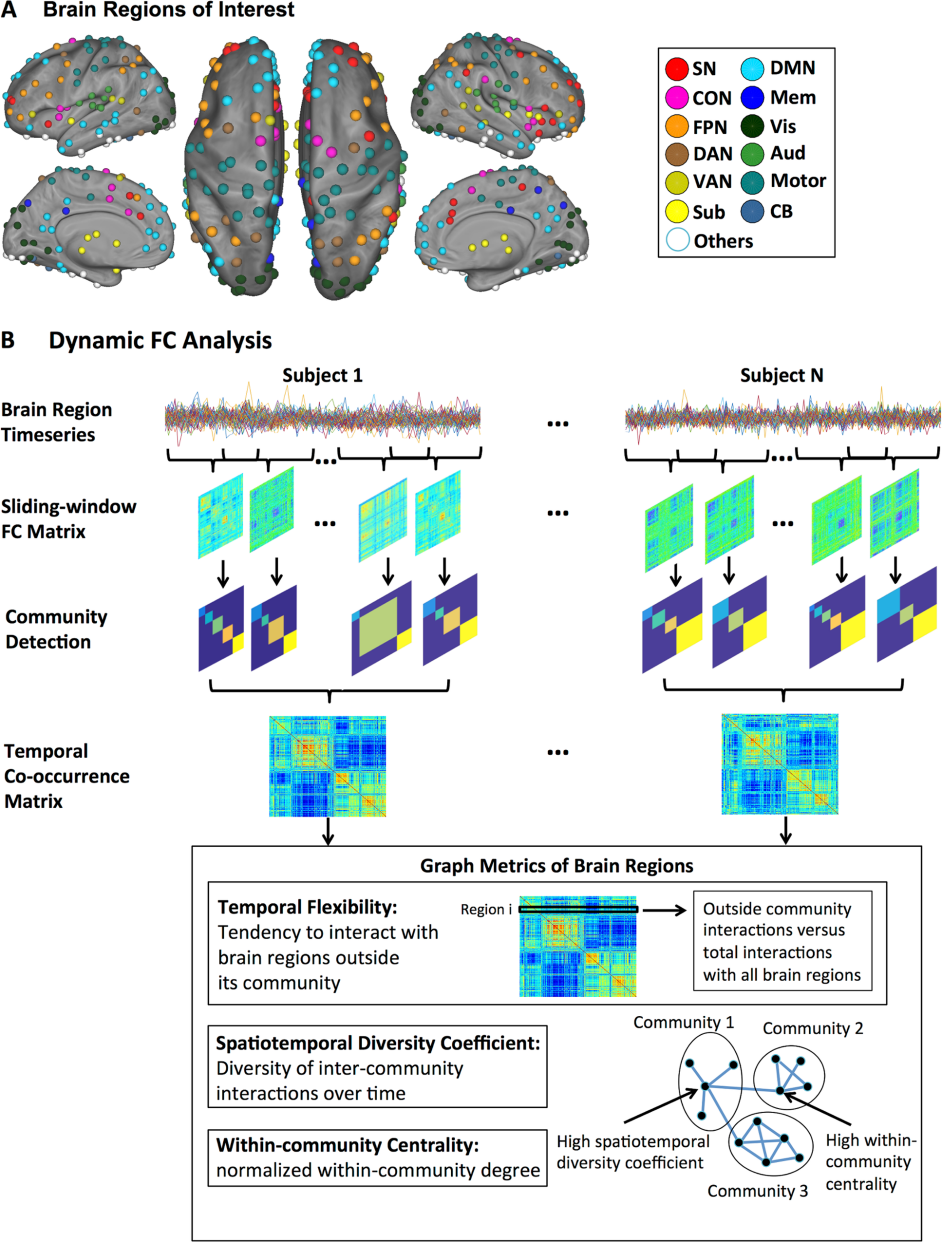
Schematic view of the main analysis steps. **(*A*)** 264 regions widely distributed across the entire brain and encompassing key (static) large-scale brain networks, including the SN, CON, FPN, DAN, VAN, subcortical, DMN, memory systems, visual, auditory, sensory-motor, and the cerebellum [3,45]. **(*B*)** Time-varying changes in the community structure of intrinsic functional connectivity were quantified using a sliding window approach. An optimized community detection algorithm was used to compute a temporal co-occurrence matrix and multiple graph metrics—(i) temporal flexibility, (ii) spatiotemporal diversity, and (iii) within-community normalized centrality—were used to characterize dynamic functional interactions between brain regions.

We analyzed dynamic functional connectivity [18,19,33] focusing on temporal excursions of each node and edge from its native (static) network configuration. Our main analysis steps are illustrated in **Fig 1.** First, we computed a temporal co-occurrence matrix, which allowed us to identify stable features associated with dynamic patterns of time-varying connectivity. Second, we quantified temporal flexibility of each node by measuring how frequently it interacts with other networks. Third, we assessed the spatiotemporal diversity of each node using an entropy-based measure of how uniformly it interacts with nodes in other networks. Finally, we investigated the relationship between temporal flexibility of each node and individual differences in cognitive flexibility using multivariate analysis with cross-validation [46]. We predicted that the SN would show highly flexible and spatiotemporally diverse dynamic interactions with other brain nodes and networks. We further hypothesized that the temporal flexibility of the SN would predict individual differences in cognitive flexibility. We show that the spatiotemporal organization of the SN is characterized by a rich repertoire of distinctive features that are behaviorally significant. Importantly, we demonstrate the test-retest reliability and robustness of all our findings across two datasets.

## Results

### Temporal Co-occurrence and Time-Varying Intrinsic Functional Interactions

**Fig 2:**
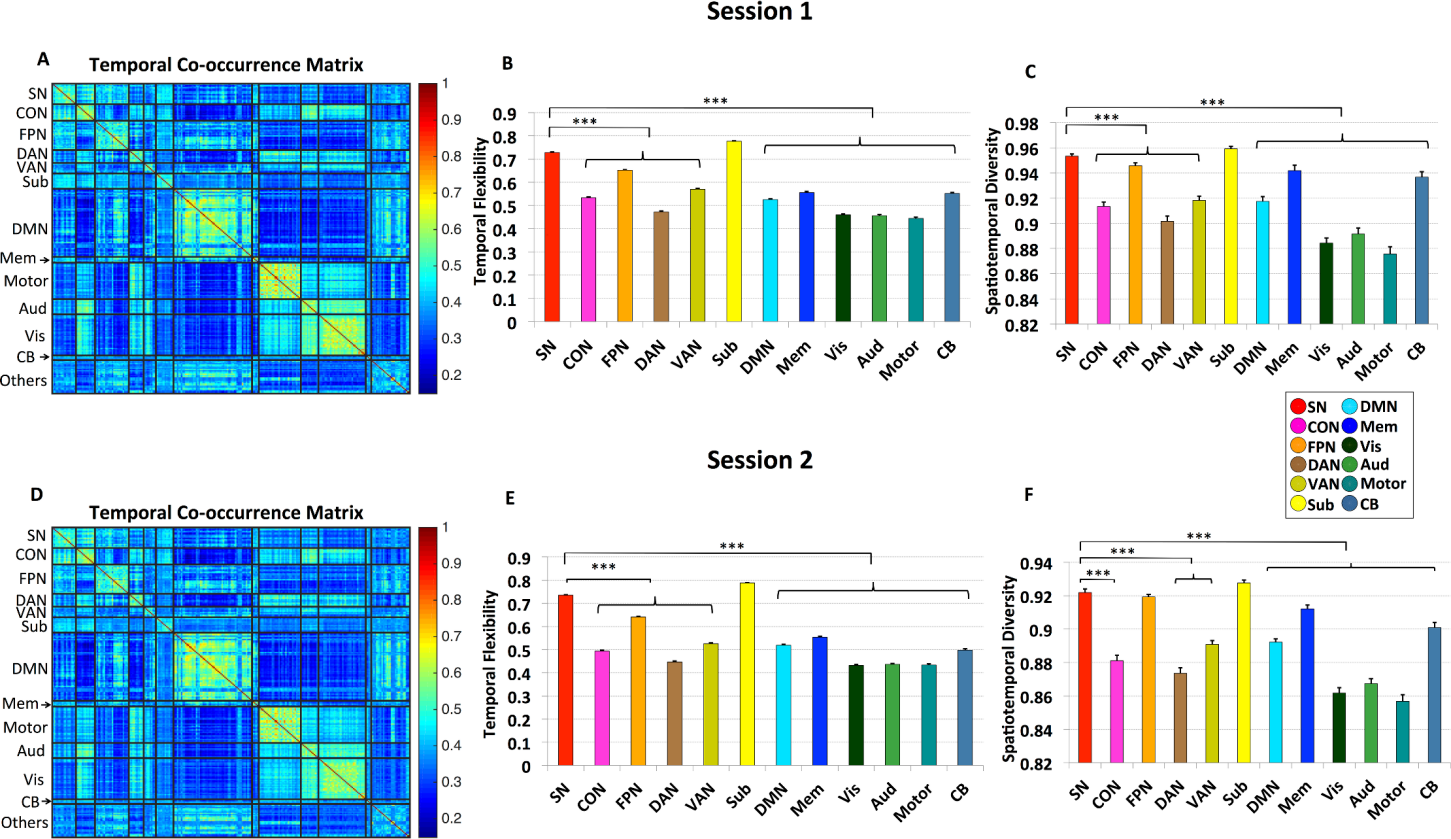
Temporal co-occurrence and time-varying intrinsic functional interactions. Panels ***A–C*** depict results from Session 1 data. **(*A*)** Temporal co-occurrence matrix for the 264 brain nodes ordered and labeled according to networks defined by previous studies [3,45]. **(*B*)** Average temporal flexibility for brain nodes in each predefined network. SN showed the highest temporal flexibility when compared to all other networks (all *ps* < 0.001), except for subcortical nodes, which displayed similar levels. **(*C*)** Average spatiotemporal diversity for brain nodes in each predefined network. SN showed the highest spatiotemporal diversity (all *ps* < 0.001), except for subcortical nodes, which displayed similar levels. Panels ***D–F*** depict corresponding results from Session 2 data. Error bars stand for standard error of the mean (SEM). (‘***’: *p* < 0.001).

To identify time-varying intrinsic functional interactions between brain regions, we used a sliding-window functional connectivity analysis [20,33] of resting-state fMRI data (**Fig 1B**). We used a set of 264 brain nodes widely distributed across the entire brain and encompassing key (static) large-scale brain networks, including SN, CON, FPN, DAN, VAN, subcortical, DMN, memory systems, visual, auditory, sensory-motor networks as well as the cerebellum (**Fig 1A**). The 264 nodes were selected from a widely used brain atlas [3,45]. We first quantified time-varying changes in the community structure of intrinsic functional connectivity using Session 1 data from 77 participants. An optimized community detection algorithm was used to compute a temporal co-occurrence matrix, in which each element measures the proportion of times that two brain nodes are part of the same community across all time windows. **Fig 2A** shows the temporal co-occurrence matrix for each pair of brain nodes labeled using networks identified in a previous study by Power and colleagues [3,45]. The temporal co-occurrence matrices from both Session 1 and 2 data have an inherent structure that is significantly different from random: the variance explained by its first principal component was significantly higher than that of random graphs with the same weight, degree, and strength distributions [47] (all *ps* < 0.001). The spatiotemporal structures of these matrices reveal brain nodes which show more frequent co-occurrence than others. Crucially, temporal co-occurrence matrices across Sessions 1 and 2 showed a similar structure (**Fig 2A and 2D**), and the average similarity between the two sessions across participants was highly significant (*r* = 0.7, *p* < 0.001). The consistency of the temporal co-occurrence matrix allowed us to uncover unique and reliable spatiotemporal features associated with the SN.

### Temporal Flexibility of Functional Interactions

Next, we used the temporal co-occurrence matrix derived above to compute the temporal flexibility of the dynamic functional interactions associated with each brain node (**Fig 1B, S1 Table, S2 Table**). Temporal flexibility is a measure of how frequently a brain region interacts with regions belonging to other communities across time. A high value of temporal flexibility would indicate that a region predominantly interacts with regions outside its own community. Brain nodes in the SN and subcortical nodes showed the highest temporal flexibility (all *ps* < 0.001, **Fig 2B**). In contrast, sensory-motor, auditory and visual nodes had the lowest temporal flexibility (**Fig 2B**). A similar pattern of results was observed in the retest dataset from Session 2 (all *ps* < 0.001, **Fig 2E**). Notably, these temporal flexibility measures capture connectivity features different from what might be predicted by static network measures (see **S1 Text** for details “**Relation between temporal flexibility and static time-averaged network measures**”).

### Spatiotemporal Diversity of Functional Interactions

We next examined brain nodes that showed the most spatiotemporally diverse intrinsic functional interactions. Here, again, we used the temporal co-occurrence matrix to compute a spatiotemporal diversity coefficient, a measure of how uniformly a brain region interacts with regions in other communities over time (**Fig 1B, S1 Table and S2 Table**). A high value for spatiotemporal diversity would indicate that interactions are more evenly distributed across communities. Crucially, brain regions that have high temporal flexibility may not have high spatiotemporal diversity if they predominately interact with brain regions in only one community; therefore, spatiotemporal diversity provides complementary information about the spatial distribution of time-varying functional connectivity. We found that brain nodes in the SN and subcortical nodes had the highest spatiotemporal diversity (all *ps* < 0.001, **Fig 2C**). Again, a similar pattern of results was observed in the retest dataset from Session 2 (all *ps* < 0.001, **Fig 2F**).

### SN Nodes Show Both High Temporal Flexibility and Spatiotemporal Diversity

**Fig 3:**
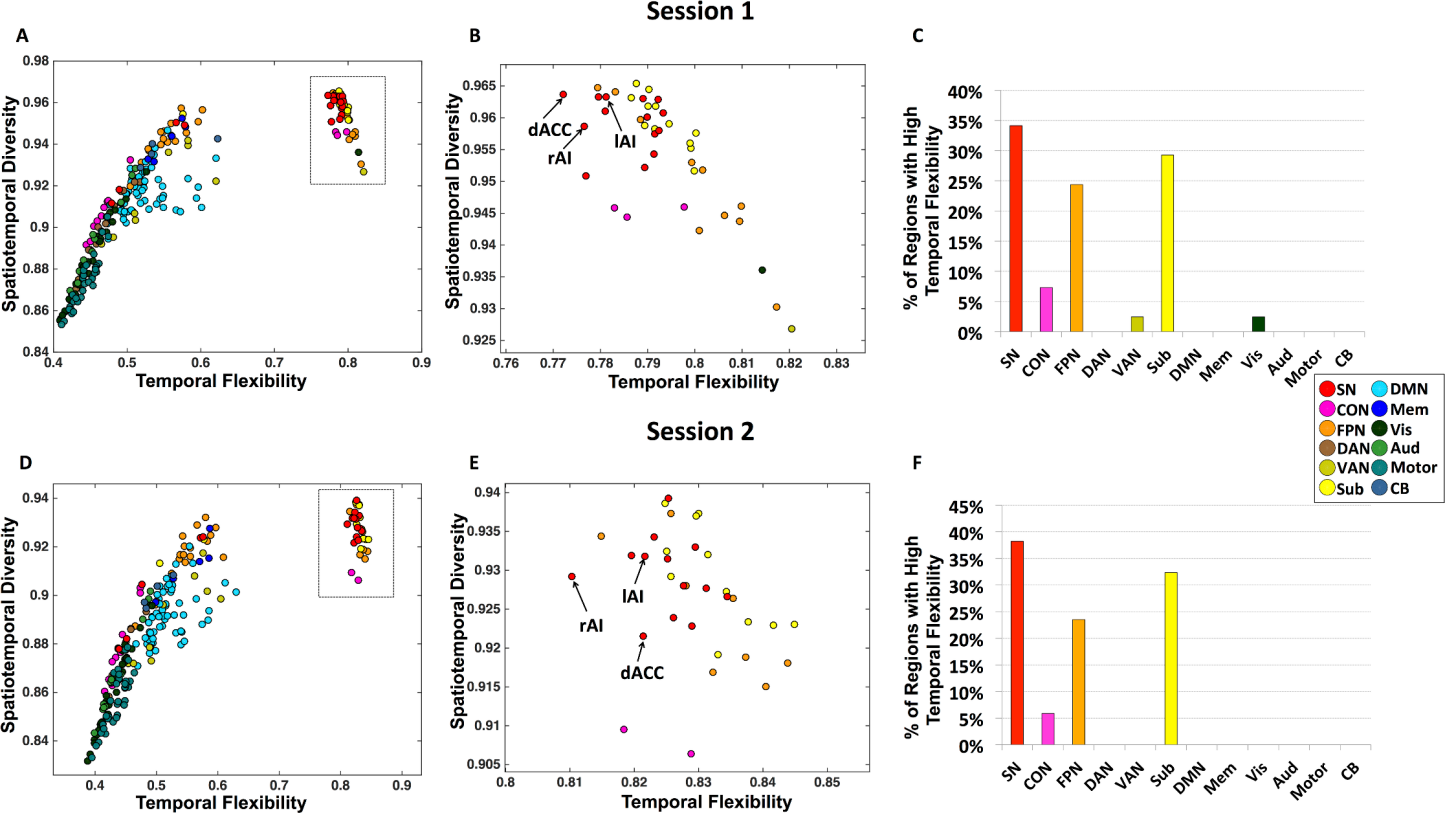
Brain regions with high temporal flexibility. Panels ***A–C*** depict results from Session 1 data. **(*A*)** Joint profile of temporal flexibility and spatiotemporal diversity identifies a cluster of brain nodes with distinctly high temporal flexibility. **(*B*)** Detailed profile of brain nodes within the cluster with high temporal flexibility (inset from panel **A**). **(*C*)** Brain nodes with high temporal flexibility are primarily from the SN, subcortical regions, and FPN, with the highest percentage belonging to the SN. Panels ***D–F*** depict corresponding results from Session 2 data.

Inspection of the joint profile of temporal flexibility and spatiotemporal diversity provided further evidence for distinct spatiotemporal properties of the SN (**Fig 3A**). We identified a cluster of nodes whose temporal flexibility-diversity profile was distinct from all other nodes. This cluster consisted primarily of brain nodes from the SN including the left and right AI and dACC (**Fig 3B**), subcortical nodes, and FPN (**Fig 3C**). To further quantify the correspondence between the results of the two sessions, we measured the number of brain nodes that showed high temporal flexibility in both sessions. We found a prominent overlap of nodes demonstrating high temporal flexibility between the two sessions: 81.3% of the brain nodes with high temporal flexibility in Session 1 also showed high temporal flexibility in Session 2; likewise, 97.5% of the brain nodes with high temporal flexibility in Session 2 showed high temporal flexibility in Session 1.

**Fig 4:**
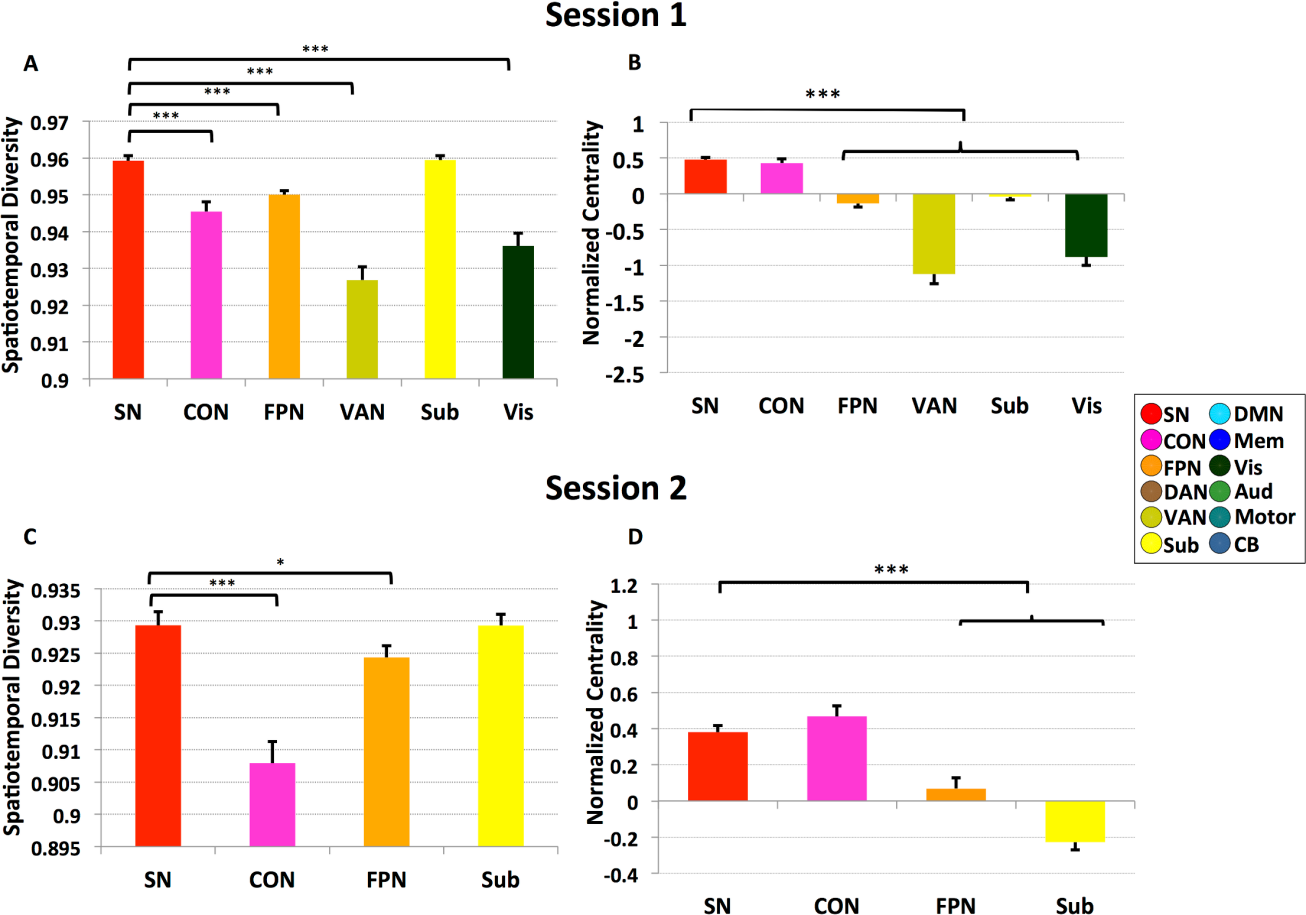
Spatiotemporal diversity and centrality of brain regions with high temporal flexibility. Panels ***A–B*** depict results from Session 1 data. **(*A*)** SN nodes have the highest spatiotemporal diversity compared to all other brain regions (all *ps* < 0.001), except for subcortical nodes, which showed similar levels. **(*B*)** SN nodes also showed the highest normalized centrality (all *ps* < 0.001), except for CON nodes, which displayed similar levels. Panels ***C–D*** depict corresponding results from Session 2 data. Error bars stand for SEM. (‘***’: *p* < 0.001, ‘*’: *p* < 0.05).

Furthermore, within this cluster of brain nodes with high temporal flexibility, SN nodes showed significantly higher spatiotemporal diversity than all other nodes (*ps* < 0.001; **Fig 4A**), except for subcortical nodes, suggesting that time-varying functional interactions of the SN are not only among the highest but also the most spatially diverse. Spatiotemporal diversity did not differ between the SN and subcortical nodes (*p* = 0.98). Notably, each of these results was also observed in Session 2 (**Fig 3D–3F; Fig 4C**). Specifically, spatiotemporal diversity of SN nodes was significantly higher than nodes implicated in cognitive control (e.g., *p* < 0.001 for nodes in CON and *p* = 0.011 for nodes in FPN) but not subcortical nodes (*p* = 0.65). Critically, these results were not observed in a dataset generated using a “noise” model obtained by applying a Fourier transform to the observed time series in each node and then adding a random phase shift sampled in the interval [0, 2π] independently to each time series and across all frequencies (see **S1 Text** “**Comparison with noise model**” for details of the methods and results). Collectively, these results indicate that key nodes of the SN show a distinct pattern of time-varying interactions characterized by the most frequent and spatially diverse interactions with other brain nodes.

### SN Nodes with High Temporal Flexibility Also Show High Centrality

Next, we examined whether, among the cluster of brain nodes that showed the high temporal flexibility (depicted in **Fig 3A–3C**for Session 1 and **Fig 3D–3F**for Session 2), SN nodes function as local information processing hubs. We found that SN and CON nodes had the highest local centrality (all *ps* < 0.001, **Fig 4B**). A similar pattern was observed in the retest dataset from Session 2: again, SN and CON nodes had the highest centrality (all *ps* < 0.001, **Fig 4D**). Finally, in both Sessions 1 and 2, SN and CON did not differ in network centrality (*p* = 0.45 and *p* = 0.27, respectively). However, SN and CON nodes differed on the spatiotemporal diversity measure, which was significantly greater in the SN for both Sessions 1 and 2 (all *ps* < 0.001; **Fig 4A and 4C**), again pointing to distinctive patterns of time-varying interactions associated with the SN (see **S1 Fig**).

### Temporal Flexibility of the SN Predicts Cognitive Flexibility

**Fig 5:**
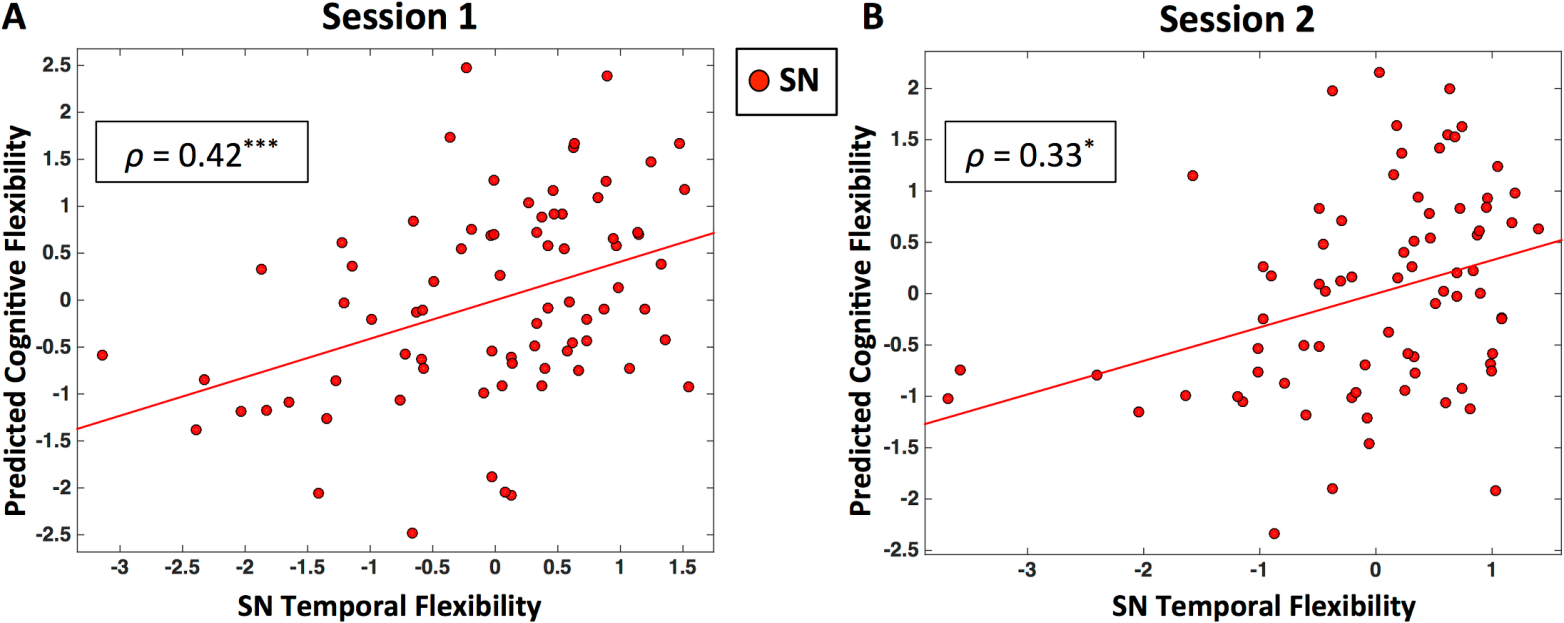
SN temporal flexibility predicts cognitive flexibility behavioral measures. Panel ***(A)*** shows results from a CCA using cross-validation on Session 1 data. Scatter plot shows predictions of individual cognitive flexibility based on SN temporal flexibility. Panel ***(B)*** shows results from Session 2 data. (‘***’: *p* < 0.001, ‘*’: *p* < 0.05).

We used canonical correlation analysis (CCA) [48] with cross-validation and prediction analysis to investigate behavioral/functional significance of our findings. The mean temporal flexibility of SN nodes with high temporal flexibility was used to predict performance measures associated with Processing Speed, Executive Function/Cognitive Flexibility, and Executive Function/Inhibition. We found that mean temporal flexibility of SN significantly predicted participants’ cognitive flexibility in Session 1 data (*ρ* = 0.42, *p* < 0.001; **Fig 5A**). Notably, this relationship was also observed in the Session 2 data (*ρ* = 0.33, *p* = 0.012; **Fig 5B**).

To examine the specificity of our SN-related brain–behavior findings, we repeated our entire CCA analyses using the mean temporal flexibility of FPN and CON nodes with high temporal flexibility. We did not find a significant relationship between FPN temporal flexibility and cognitive flexibility (*ρ* = 0.16, *p* = 0.15 for Session 1 data; *ρ* = 0.21, *p* = 0.08 for Session 2 data), nor a significant relationship between CON temporal flexibility and cognitive flexibility (*ρ* = 0.13, *p* = 0.16 for Session 1 data; *ρ* = 0.01, *p* = 0.44 for Session 2 data). We also found no relation between temporal flexibility of DAN and VAN and cognitive flexibility (*ps* > 0.1), further demonstrating the specificity of our SN related brain–behavior findings. Because subcortical nodes also showed high levels of temporal flexibility, we repeated the same analysis for these nodes. There was no significant relation between temporal flexibility of the subcortical nodes and cognitive flexibility (*ρ* = 0.22, *p* = 0.07 for Session 1 data; *ρ* = 0.23, *p* = 0.05 for Session 2 data). Finally, we also did not find any relationship between static connectivity measures and cognitive flexibility (all *ps* > 0.05) (see **S1 Text** “**Static time-averaged network measures do not predict cognitive flexibility**” for details).

## Discussion

We investigated the dynamic spatiotemporal organization of the SN using high temporal-resolution fMRI data from the open-source HCP. Novel network analysis uncovered distinctive, robust, and behaviorally-relevant properties of time-varying connectivity associated with the SN. Several findings are noteworthy with respect to critical unaddressed questions regarding dynamic functional interactions of large-scale brain networks and the SN in particular. First, dynamic connectivity analysis revealed that the SN together with subcortical nodes have the highest level of temporal flexibility in the brain, significantly higher than FPN, CON, DAN, and VAN. Second, dynamic functional interactions of the SN were also among the most spatially diverse in the brain. In contrast, connectivity of the sensory and motor networks was much less flexible and less diverse (**Figs 2 and 3**). Third, SN nodes maintained a consistently high level of centrality over time, suggesting that the SN is a hub for facilitating flexible cross-network interactions. Fourth, time-varying connectivity profiles of the SN were distinct from all other prefrontal control systems. Fifth, temporal flexibility of the SN, but not the FPN, CON, DAN, or VAN, predicted individual differences in cognitive flexibility. Importantly, each of these results was replicated in data from a second session in the same group of participants, demonstrating the robustness of our findings. Taken together, our findings provide important new insights into the highly flexible yet stable organization of the SN and reveal how its transient dynamic interactions with other large-scale networks support functional integration while maintaining a stable segregated core. As elaborated below, these findings are noteworthy in the context of large-scale brain organization and, specifically, within the context of the SN and other networks important for implementing cognitive control in the human brain [13,17].

### Dynamic and Consistent Spatiotemporal Brain Organization

Most previous studies of intrinsic functional brain connectivity have focused on the static organization of brain networks and, under assumptions of stationarity, have consistently identified the SN and several key brain networks involved in cognitive control and attention [1–3]. We used novel dynamic graph-theoretical techniques to investigate dynamic functional interactions between 264 widely distributed brain regions implicated in a wide range of cognitive paradigms [3,45]. Dynamic changes in connectivity were assessed in the context of a previously defined framework of twelve brain networks, which include, in addition to the SN, other cognitive control systems such as the CON, FPN, DAN, and VAN [3,45]. An important question our study addresses at the outset is whether it is possible to identify stable features associated with dynamic patterns of time-varying connectivity. We found that each of the brain networks previously identified using static network analysis not only displayed strong within-network interactions, consistent with the expected pattern of segregated networks, but also prominent time-varying cross-network interactions, reflecting integration across functional networks [18,20,33]. A key finding here concerns the temporal co-occurrence matrix, which captures statistical properties of time-varying connectivity across functional networks. Each element of this matrix measures the proportion of time that two brain regions are part of the same network. Despite highly variable time-varying connectivity across brain regions and participants, the temporal co-occurrence matrix, far from being random, displayed a structure that reflected a balance between functional segregation and integration. Importantly, the temporal co-occurrence matrix also displayed a high level of similarity across Sessions 1 and 2 (**Fig 2A and 2D**), pointing to stable features associated with time-varying connectivity. Such a consistent pattern of spatiotemporal brain organization allowed us to uncover unique and reliable dynamic properties of the SN.

### SN Is a Highly Flexible System with the Most Diverse Interactions

Our analysis of time-varying connectivity provides fundamental insights into the functional organization of the SN. Analysis of the evolution of dynamic connectivity patterns revealed that the SN has properties that distinguish it from other functional brain systems, including all other brain systems that have been implicated in various aspects of cognitive control. SN nodes were the most temporally flexible and had disproportionally higher interactions with other networks, a finding that was robust across the two sessions (**Fig 2B and 2E**). SN nodes were more flexible than nodes of the FPN, VAN, DAN, and CON, other key prefrontal cortex systems that have been widely implicated in cognitive control [5,11,27]. Only subcortical nodes showed a similarly high level of temporal flexibility. Critically, the same SN nodes that showed high temporal flexibility also showed the most spatially diverse time-varying connectivity, indicating that this network, and its AI and dACC nodes in particular, has the most frequent as well as the most varied interactions with other functional brain systems. Here, again, SN nodes showed the highest spatiotemporal diversity compared to nodes of the FPN, VAN, DAN, and CON, suggesting that time-varying functional interactions of the SN are not only among the highest but also most spatially varied (**Fig 2C and 2F**). Notably, each of these results was also observed in Session 2 data. Collectively, these results indicate that key SN nodes show a distinct pattern of time-varying interactions characterized by the most frequent and spatially diverse interactions with other brain regions.

### SN Is a Unique Hub for Driving Cross-Network Interactions

The joint profile of temporal flexibility and spatiotemporal diversity provided further evidence for distinct spatiotemporal properties of the SN. Of particular interest is a cluster of regions whose temporal flexibility-diversity profile was distinct from all other regions (**Fig 3A and 3D**). Within this cluster, SN nodes, including the right and left AI and dACC, showed a distinctive pattern characterized by the highest flexibility and spatiotemporal diversity among all brain regions (**Fig 4A and 4C**). This cluster consisted primarily of nodes from just three functional systems: the SN, subcortical regions, and FPN, with the largest set of nodes belonging to the SN (**Fig 3C and 3F**). Another important feature of the SN, which distinguishes it from other control systems, including the FPN, VAN, DAN, and CON, is that it maintains a consistently high level of node centrality over time, suggesting that the SN is a hub for facilitating flexible cross-network interactions. Only CON nodes showed similar levels of centrality as the SN. However, SN nodes were unique in that they had a significantly higher temporal flexibility and spatiotemporal diversity than CON nodes in data from both Sessions 1 and 2, pointing to a distinct spatiotemporal organization of SN characterized by distinctively high temporal flexibility, high diversity, and high local centrality (see **S1 Fig**). Our findings provide novel evidence for a dynamic functional basis for dissociation of the SN from all other brain networks and functional systems, including other control networks, memory, DMN, and sensorimotor networks.

### Functional Implications of a Dynamic SN

The unique functional properties of the SN identified in the present study have important implications for understanding human brain function. Critically, the high temporal flexibility and spatiotemporal diversity of the SN make it uniquely positioned to facilitate interactions with multiple functional systems. Consistent with this view, functional neuroimaging studies have consistently shown that the AI and dACC nodes of the SN are among the most highly and flexibly activated brain regions across a wide range of cognitive tasks [5,11,23]. Prominent models of cognitive and attentional control in the human brain have implicated multiple functional systems, including the SN in detection of and orientation to salient events [5,11,23] and the VAN and DAN in bottom-up and top-down attention [49], while other dual control models have highlighted the role of the FPN and CON in adaptive and stable task control [25,50]. Although the dynamic roles of these systems are still not well understood, it is noteworthy that the dynamic properties of the SN, as characterized in the present study, distinguish it from other cognitive control systems. The high temporal flexibility and spatially diverse connectivity of the SN along with the unique presence of fast conducting von Economo neurons in great apes and humans [51], which are uniquely expressed in the AI and dACC, make the SN ideally positioned to initiate adaptive control processes. Consistent with this view, several recent studies have shown that the SN displays distinctively fast responses to external and internal salient events, including errors [4,7,52], and that disruptions to this network impair task switching [32,53].

Furthermore, multiple studies using several different computational approaches have found that that the SN, its AI node in particular, has strong causal influences on other fronto-cingulate-parietal regions during tasks that require adaptive cognitive control [4,7,9,10]. Task-based functional neuroimaging studies have also identified a prominent role for the SN in switching between functional systems [4,9,24,29,53], and a triple network model, anchored in the SN, posits that this network plays a crucial role in switching between functional systems and facilitating access to attentional and cognitive resources [17]. The model predicts that a flexible functional organization of the SN is crucial for initiating cognitive control processes [17,54]. Consistent with this model, we found that the dynamic temporal flexibility and spatiotemporal organization of the SN predicted individual differences in cognitive flexibility. Importantly, this brain–behavior relation was observed in both datasets and was specific to the SN, as FPN and subcortical nodes did not have such a relation despite high levels of temporal flexibility (**Fig 2B and 2E**). The present findings provide novel support for the triple-network model and highlight a previously unknown dynamic spatiotemporal organization that underlies the ability of the SN to flexibly engage with a diverse set of brain regions. Finally, the unique dynamic functional properties of the SN also suggest that disruptions to the spatiotemporal organization of this key network can have a significant detrimental effect on a wide range of cognitive, social and affective functions [13,29,32,53]. Consistent with this view, SN dysfunction has now been demonstrated to be a prominent feature of a large number of psychiatric and neurological disorders, including autism, schizophrenia, and dementia [14–16,55–57].

### Contrasting Temporal Dynamics of the SN and FPN

Although some of the FPN nodes demonstrated high temporal flexibility, on average the temporal flexibility of the FPN nodes was significantly lower than the SN in both the test and retest data. Furthermore, as noted above, in contrast to the SN, temporal flexibility of the FPN did not predict individual differences in cognitive flexibility. These findings are noteworthy in the context of a previous study, which found that brain-wide functional connectivity pattern of the FPN shifts more, from rest, than those of other networks across a variety of task states [58]. It should, however, be noted that changes between task and rest were assessed using static connectivity measures. Because the SN has been shown to function as dynamic causal signaling hub during tasks involving attention and inhibitory control [4,6,9], it is unlikely that these differences solely reflect distinctions between intrinsic and task states. Rather, we propose that the nature of flexible hubs may shift dynamically over time with more flexible and rapid processing initially facilitated by the SN to more sustained processing sustained over a longer time period by the FPN. Studies examining dynamic connectivity changes associated with cognitively demanding tasks are needed to address this question.

## Conclusion

Our findings highlight several distinctive, robust, and reliable functional properties of the SN. Crucially, the SN has a unique spatiotemporal organization in the human brain characterized by high temporal flexibility, spatiotemporal diversity, and node centrality. Notably, time-varying connectivity profiles of the SN are distinct from all other cognitive control systems, including the FPN, DAN and VAN. Our findings provide fundamental new insights into how the dynamic functional architecture of the SN uniquely positions it to facilitate interactions with multiple functional systems and thereby support a wide range of cognitive processes. Finally, our findings and computational methods provide a template for future investigations of SN dysfunction in psychiatric disorders.

## Materials and Methods

Data acquisition for the HCP was approved by the Institutional Review Board of The Washington University in St. Louis (IRB # 201204036), and all open access data were deidentified.

### Functional MRI Data: Session 1

Minimally preprocessed resting-state fMRI data were obtained from the HCP under the Q1-Q6 Data Release. Seventy-eight individuals (Session 1, left-right encoded, age: 22–35, 28 males) were selected from 500 individuals based on the following criteria: (1) individuals are unrelated; (2) range of head motion in any translational direction is less than 1 mm; (3) average scan-to-scan head motion is less than 0.2 mm, and (4) maximum scan-to-scan head motion is less than 1 mm. One subject was excluded due to artifacts. For each individual, 1,200 frames were acquired using multiband, gradient-echo planar imaging with the following parameters: RT, 720 ms; echo time, 33.1 ms; flip angle, 52°; field of view, 280 × 180 mm; matrix, 140 × 90; and voxel dimensions, 2 mm isotropic. During scanning, each individual was eye-fixated on a projected crosshair on the screen. See **S3 Table** for the basic demographic information of the participants.

### fMRI Dataset: Session 2

Resting-state fMRI data from the second session of the same 77 individuals (left-right encoded) were used for test-retest reliability of findings form Session1.

### Data Processing

The same processing steps and network analyses were applied to Session 1 and Session 2 data (**Fig 1**). Spatial smoothing with a Gaussian kernel of 6 mm FWHM was first applied to the minimally preprocessed data to improve signal-to-noise ratio as well as anatomy correspondence between individuals. A multiple linear regression approach with 12 realignment parameters (3 translations, 3 rotations, and their first temporal derivatives) was applied to the smoothed data to reduce head-motion-related artifacts. To further remove physiological noise, an independent component analysis (ICA) was applied to the preprocessed data using Melodic ICA version 3.14. ICA components for white matter and cerebrospinal fluid (CSF) were first identified and their corresponding ICA time-series were then extracted and regressed out of the preprocessed data.

### ROI Timeseries

Large-scale networks were identified based on an atlas of 264 brain nodes of interest [3,45]. The set of 264 brain nodes were widely distributed across the entire brain and encompass key large-scale brain networks of interest, including SN, CON, FPN, DAN, VAN, subcortical, DMN, memory systems, visual, auditory, sensory-motor networks as well as the cerebellum [3,45]. For each individual, mean signals from each of the 264 nodes were extracted, and the first 20 frames were discarded to minimize nonequilibrium effects in fMRI signal. The resulting time-series were further high-pass filtered (*f* > 0.008 Hz) to remove low frequency signals related to scanner drift. Subsequent network analysis was performed using the Brain Connectivity Toolbox [47].

### Modularity Analysis

We used graph-theoretical and community detection techniques to investigate static and time-varying connectivity between the 264 nodes [20,59,60]. Community detection was used to determine the optimal modular structure within the functional connectivity matrix by grouping nodes into nonoverlapping communities or modules that maximize intramodular connectivity and minimize intermodular connectivity [61]. We used the complete unthresholded, signed, and weighted connectivity matrix to avoid use of arbitrary thresholds, overcoming limitations of previous studies [3,45]. The Louvain algorithm [62] implemented in the Brain Connectivity Toolbox [47] was used to detect community structure in both the static and time-varying connectivity matrices. This algorithm optimizes a quality function Q*, defined as the difference between the observed intramodular connectivity and the intramodular connectivity expected by chance, while penalizing assignment of nodes with negative correlations to the same community [62]. The Louvain algorithm automatically determines the number of underlying communities, and the resulting community structure is characterized by high positive and low negative connectivity within each community.

It should be noted that this community structure was based on an unbiased weighted connectivity matrix, i.e., we did not impose an arbitrary threshold on the connectivity matrix. One commonly adopted and critical step in such analyses is to create a binary adjacency matrix by thresholding an association matrix (e.g., cross-correlation between brain nodes) at an arbitrary value [3,59]. However, the use of such arbitrary thresholds is problematic, as it can lead to different levels of network sparsity and highly biased estimates of community structure. Critically, the use of such thresholds is highly problematic for investigating time-varying connectivity as the optimal threshold at each time point is not known and can further bias community detection. Our approach here overcomes these limitations.

### Static Functional Connectivity Analysis

For each individual, the static functional connectivity between nodes was first computed using Pearson correlations and the entire nodal time-series. The resulting correlation values were then z-transformed and averaged across individuals, representing group-averaged functional connection strength. Modularity analysis was then performed and randomly initialized 100 times to determine the optimal static modular structure within this group-averaged functional connectivity matrix. These static communities spanning the whole brain were used as a reference community structure for computing the node-level graph-theoretical measures, as described in the sections below.

### Time-Varying Functional Connectivity Analysis

Dynamic functional connectivity between nodes was computed using a sliding window with a gap of one TR between windows and exponentially decaying weights applied to each time point within a window [20]. The exponentially decaying weights were computed as:

$$w_{t}=w_{0}e^{\left(t-T\right)/\theta },\;t=1,\cdots ,T,$$

where *w*_0_ = (1 − *e*^−1/*θ*^)/(1 − *e*^−*T*/*θ*^), *t* is the *t*^*th*^ time point within the sliding window, *T* is the sliding window length, and the exponent *θ* controls the influence from distant time points. *θ* was set to a third of the window length, consistent with previous studies [20]. For the main results, we used a window length of 40 s used in the previous study [33]. We also used a window length of 20 s and found that our results were robust to window length (see **S1 Text** for details, “**Temporal dynamics on a shorter time-scale**”). Within each time window, we constructed a functional connectivity matrix by computing the weighted Pearson correlation between time-series of any two nodes *x*_*t*_ and *y*_*t*_ as:

$$r_{w}=\frac{\sum_{t=1}^{T}w_{t}(x_{t}-\bar{x})(y_{t}-\bar{y})}{\sqrt{\sum_{t=1}^{T}w_{t}{\left(x_{t}-\bar{x}\right)}^{2}}\sqrt{\sum_{t=1}^{T}w_{t}{\left(y_{t}-\bar{y}\right)}^{2}}},$$

where $\bar{x}=\frac{\sum_{t=1}^{T}w_{t}x_{t}}{T}$ and $\bar{y}=\frac{\sum_{t=1}^{T}w_{t}y_{t}}{T}$. The resulting weighted Pearson correlation matrix was z-transformed for subsequent analysis.

### Dynamic Functional Network Analysis—Temporal Co-occurrence Matrix

To investigate dynamic interactions between brain nodes, we first performed modularity analysis (as described in the section on modularity analysis above) on the functional connectivity matrix within each sliding window for each participant. The modularity analysis was randomly initialized 100 times to determine the optimal community structure within each sliding window. The community structure within each sliding window was used to construct an adjacency matrix *A*_*ijtk*_ for each participant, such that *A*_*ijtk*_ = 1 if node *i* and node *j* are in the same community at time window *t* for individual *k*, otherwise *A*_*ijtk*_ = 0. The temporal co-occurrence matrix was thus computed as the temporal mean of the adjacency matrix: $C_{ijk}=\frac{\sum_{t=1}^{T}A_{ijtk}}{T}$, similar to the module-allegiance measure in previous studies [38–40]. Each element measures the proportion of times that two brain regions are part of the same community. A high value indicates that the two corresponding brain regions coparticipate in the same community more frequently.

### Node-Level Metrics of Dynamic Functional Connectivity

Based on the temporal co-occurrence matrix *C*_*ijk*_ and the static modular organization of nodes, we characterized the dynamic spatiotemporal properties of each node using three measures: (1) temporal flexibility, (2) spatiotemporal diversity, and (3) within-community centrality.

(1) The temporal flexibility of node *i* and participant *k* was computed as:

$$f_{ik}=\frac{\sum_{j\notin u_{i}}C_{ijk}}{\sum_{j\neq i}C_{ijk}},$$

where *C*_*ijk*_ is the temporal co-occurrence matrix for individual *k*, and *u*_*i*_ is the community to which node *i* belongs, $\sum_{j\notin u_{i}}C_{ijk}$ measures the frequency with which node *i* engages in interactions with nodes outside its native community. ∑_*j* ≠ *i*_ *C*_*ijk*_ measures the total interactions with all nodes. Thus, temporal flexibility captures the tendency of each node to deviate from its own native community and interact with outside nodes. Our measure of temporal flexibility is similar to the one used by Bassett and colleagues [37,39,40,63], but there are some key differences. Crucially, we did not impose any constraints of temporal coupling across adjacent temporal windows. Consequently, unlike multilayer community detection algorithms [63], our approach did not require estimation of additional free parameters.

(2) The spatiotemporal diversity of node *i* and participant *k* was computed in the same way as the diversity coefficient [64], except that the magnitude of links between nodes quantifies time-varying changes in the intrinsic community structures (temporal co-occurrence). Specifically, the spatiotemporal diversity was computed as:

$$h_{ik}=-\frac{1}{log(m)}\sum_{u\in M}p_{ik}(u)logp_{ik}(u),$$

where $p_{ik}(u)=\frac{s_{ik}(u)}{s_{ik}}$, *s*_*ik*_ is the degree/strength of node *i* among all communities for participant *k*, *s*_*ik*_(*u*) is the degree/strength of node *i* in community *u* for participant *k*, *m* is the total number of communities, and *M* is the set of communities. Nodes with high spatiotemporal diversity scores are those that have relatively spatially varied distribution of time-varying interactions with all communities and are putative loci for integrating information between communities.

(3) The within-community normalized centrality of node *i* and participant *k* was computed as:

$$z_{ik}=\frac{s_{ik}(m_{i})-\bar{s_{k}}(m_{i})}{\sigma^{s_{k}(m_{i})}},$$

where *z*_*ik*_ is the within-community normalized centrality for node *i* and participant *k*, *m*_*i*_ is the community that contains node *i*, *s*_*ik*_(*m*_*i*_) is the degree/strength for node *i* within the community *m*_*i*_ for participant *k*, $\bar{s_{k}}(m_{i})$ is the mean degree/strength of all nodes within community *m*_*i*_ for individual *k*, and $\sigma^{s_{k}(m_{i})}$ is the standard deviation of node degree/strength within the community *m*_*i*_ for participant *k*. The within-community normalized centrality quantifies the centrality of a node within its own community. Nodes with high centrality are local core information processing hubs.

### Behavioral Measures of Cognitive Flexibility

We identified three measures of cognitive flexibility that were administered to each HCP participant. These measures were part of the HCP NIH toolbox, a multidimensional set of brief measures assessing cognitive function. The toolbox was designed to create a standard set of measures that could be used as a common metric across diverse study designs and settings [65,66]. The three measures were related to Executive function, Attention and Processing Speed components of the toolbox: Processing Speed from the Pattern Completion Processing Speed Test, Executive Function/Cognitive Flexibility from the Dimensional Change Card Sort Test, and Executive Function/Inhibition from the Flanker Task [44,65]. See **S3 Table** for behavioral measures of cognitive flexibility for each participant.

### Brain–Behavior Analysis

The relation between dynamic connectivity measures and cognitive flexibility was examined using CCA and cross-validation procedures. CCA is a statistical method for examining the relationships between two multivariate sets of variables [48] and has been shown to be a powerful tool for investigating brain–behavior relationships [46]. CCA finds the optimal linear combination of subjects’ multivariate behavioral measures that maximize the relation between behavioral and brain measures. CCA prediction analysis was performed using 4-fold cross-validation. Participants were first randomly divided into four folds. Behavioral and brain data in three folds were used for training while the remaining fold was used for testing. CCA analysis was used to find the optimal combination weights of the behavioral and brain measures. The weights were then applied to data from the test fold to obtain the prediction between behavioral and brain measures. These procedures were repeated all test datasets, and the correlation between the predicted behavioral and brain measures were computed using Spearman correlation. To test whether the correlation was significant, we used a permutation test in which brain measures were randomized across participants to create 10,000 null datasets. For each null dataset, we repeated the same cross-validation analysis and formed an empirical null distribution for determining the significance of the observed correlation.

## Supporting Information

S1 Data Co-occurrence matrices. (MAT)

S2 Data Data for main and supporting figures. (XLSX)

S1 Fig Visualization of spatiotemporal dynamics measures for regions with high temporal flexibility. Panels ***A–B*** depict results from Session 1 data. **(*A*)** SN nodes, especially rAI and dACC, are distinguished from other nodes with high temporal flexibility by their profile of high spatiotemporal diversity and centrality. **(*B*)** Force-directed graph representation of the relation between SN nodes and other nodes using the Kamada–Kawai algorithm [67], based on the three graph theoretical measures shown in panel A. SN nodes, especially rAI and dACC, are separated from other nodes. Panels ***C–D*** depict corresponding results for Session 2 data.
(TIF)

S2 Fig Time-varying dynamic connectivity assessed using a 20 s sliding window. Panel **A–B** depicts results for Session 1 data. **(*A*)** The joint profile of temporal flexibility and spatiotemporal diversity identifies a cluster of brain nodes with distinctly high temporal flexibility. **(*B*)** Average temporal flexibility for brain nodes in each predefined network. SN showed the highest temporal flexibility when compared to all other networks (*ps* < 0.001), except for subcortical nodes, which displayed similar levels. Panels **C–D** depict the corresponding results for Session 2 data.
(TIFF)

S3 Fig Relation between dynamic temporal flexibility and static functional connectivity measures. Panels **A–B** depict results from Session 1 data. ***(A)*** Relationship between temporal flexibility and static participation coefficient. ***(B)*** Relationship between temporal flexibility and static node strength. Panels ***C–D*** depict corresponding results for Session 2 data.
(TIFF)

S1 Table MNI coordinates, temporal flexibility and spatiotemporal diversity of all 264 nodes, Session 1 data. (XLSX)

S2 Table MNI coordinates, temporal flexibility and spatiotemporal diversity of all 264 nodes, Session 2 data. (XLSX)

S3 Table HCP participant identifier and demographics. (XLSX)

S1 Text Additional supporting results sections. (DOCX)

## Abbreviations

AI: anterior insula
CCA: canonical correlation analysis
CON: cingulate–opercular network
CSF: cerebrospinal fluid
dACC: dorsal anterior cingulate cortex
DAN: dorsal attention network
DMN: default mode network
FPN: frontoparietal network
HCP: Human Connectome Project
ICA: independent component analysis
SEM: standard error of the mean
SN: salience network
VAN: ventral attention network
