# Supplementary figures and images for: Distinct Global Brain Dynamics and Spatiotemporal Organization of the Salience Network
Source: PLoS Biol. 2016 Jun 7;14(6):e1002469. doi: 10.1371/journal.pbio.1002469 (PMC4896426; doi:10.1371/journal.pbio.1002469)

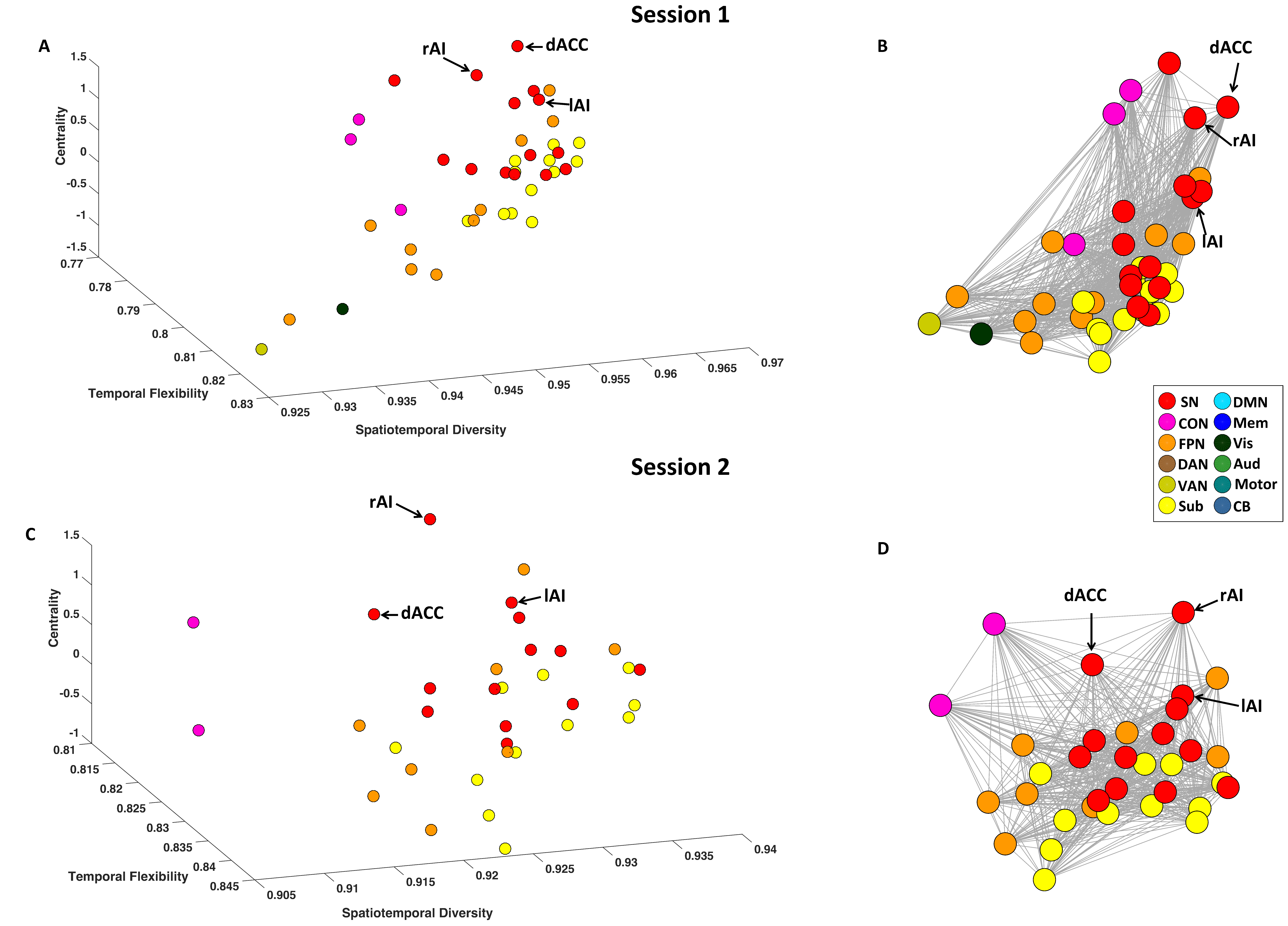

Supplement: S1 Fig — Panels A–B depict results from Session 1 data. (A) SN nodes, especially rAI and dACC, are distinguished from other nodes with high temporal flexibility by their profile of high spatiotemporal diversity and centrality. (B) Force-directed graph representation of the relation between SN nodes and other nodes using the Kamada–Kawai algorithm [67], based on the three graph theoretical measures shown in panel A. SN nodes, especially rAI and dACC, are separated from other nodes. Panels C–D depict corresponding results for Session 2 data. (TIF) [file pbio.1002469.s003.tif]

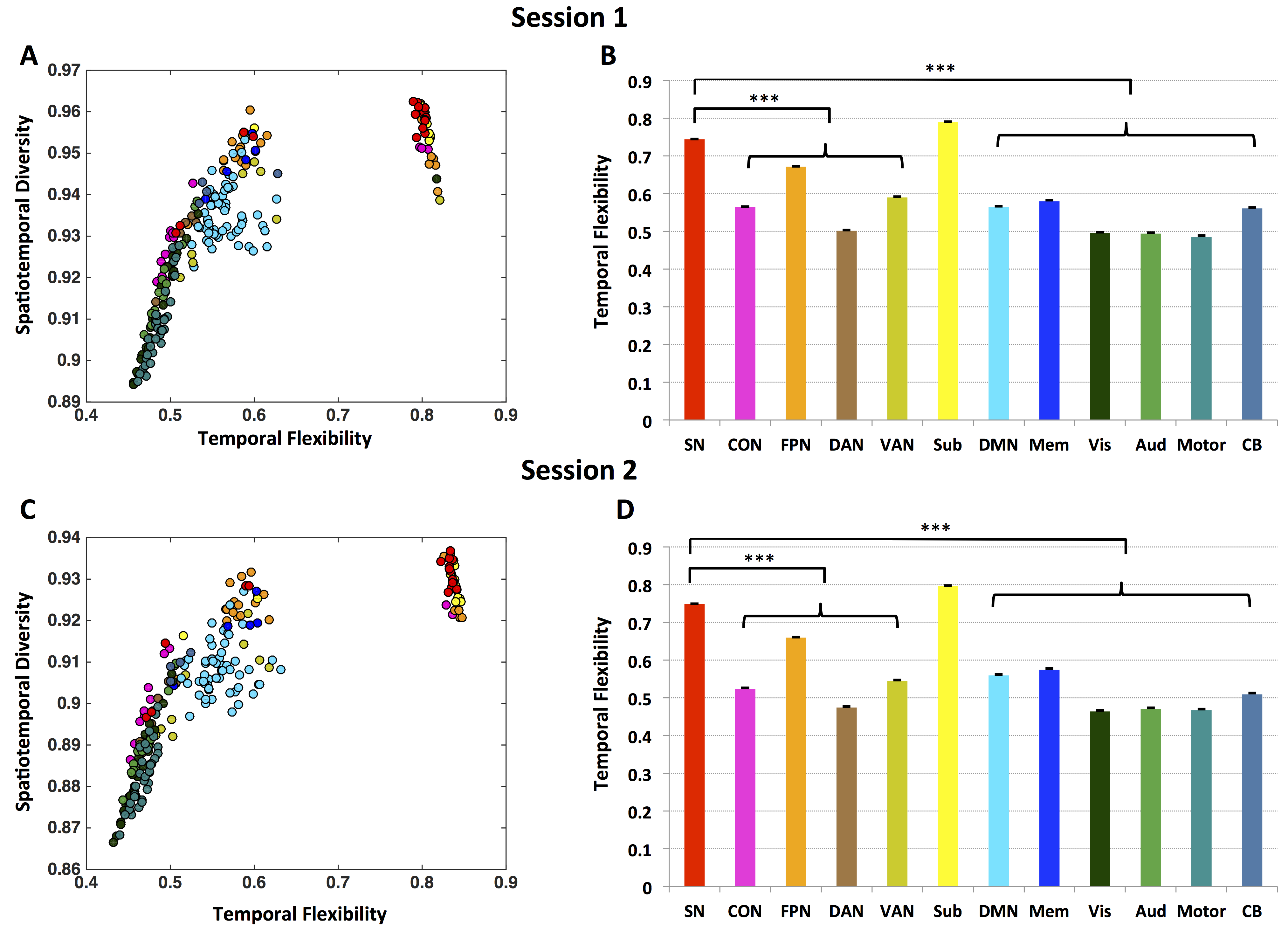

Supplement: S2 Fig — Panel A–B depicts results for Session 1 data. (A) The joint profile of temporal flexibility and spatiotemporal diversity identifies a cluster of brain nodes with distinctly high temporal flexibility. (B) Average temporal flexibility for brain nodes in each predefined network. SN showed the highest temporal flexibility when compared to all other networks (ps < 0.001), except for subcortical nodes, which displayed similar levels. Panels C–D depict the corresponding results for Session 2 data. (TIFF) [file pbio.1002469.s004.tiff]

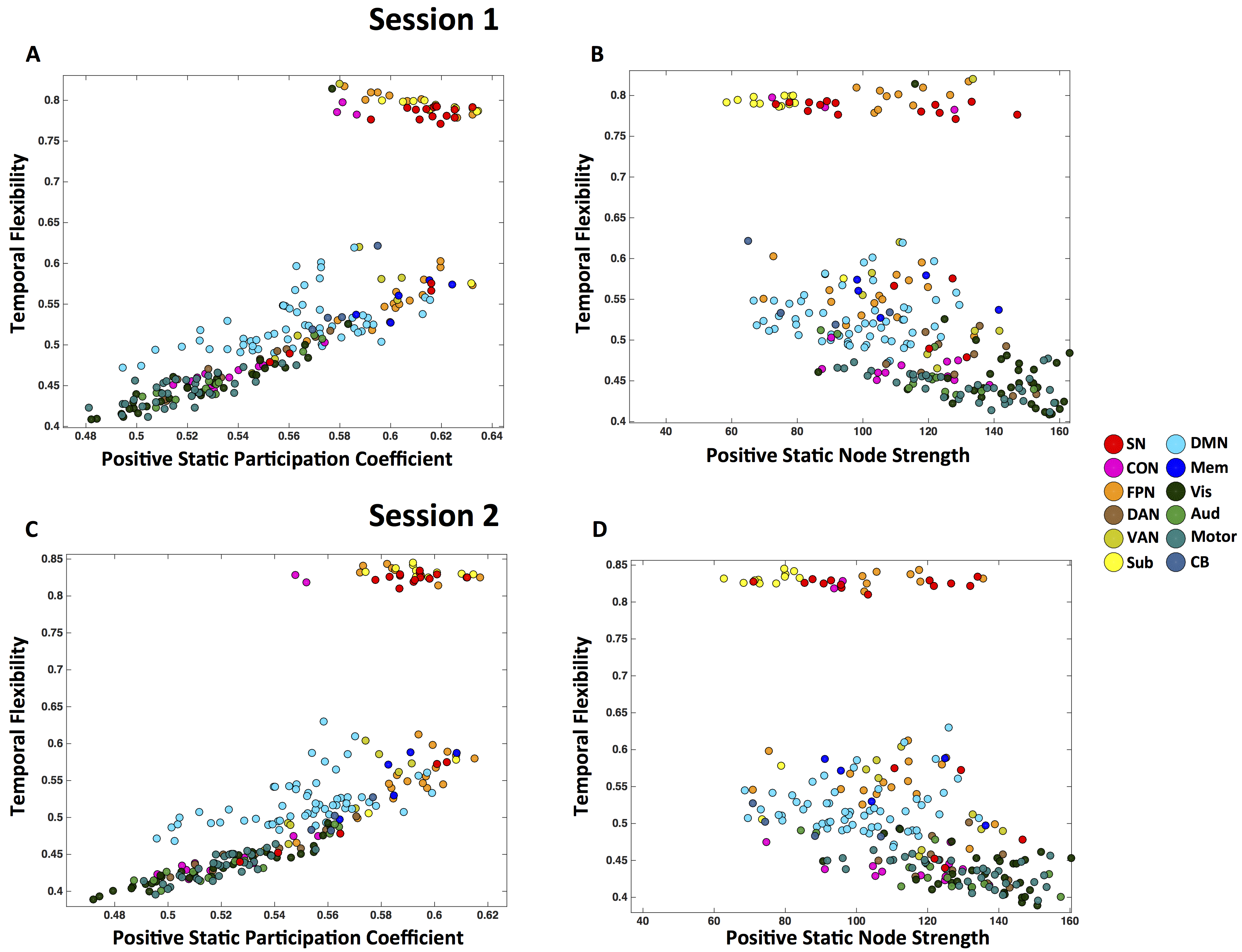

Supplement: S3 Fig — Panels A–B depict results from Session 1 data. (A) Relationship between temporal flexibility and static participation coefficient. (B) Relationship between temporal flexibility and static node strength. Panels C–D depict corresponding results for Session 2 data. (TIFF) [file pbio.1002469.s005.tiff]
