# Supplementary material for: Distinct Global Brain Dynamics and Spatiotemporal Organization of the Salience Network
Source: PLoS Biol. 2016 Jun 7;14(6):e1002469. doi: 10.1371/journal.pbio.1002469 (PMC4896426; doi:10.1371/journal.pbio.1002469)
Supplement: S1 Text — (DOCX) [file pbio.1002469.s009.docx]

**Supporting Information**

**I. Supplementary Results and Discussion**

***Uniqueness of the Salience Network***

The high temporal flexibility nodes of the SN have unique properties that distinguish them from those in the CON, FPN and subcortical regions (**S1** **Fig A-D)**. Compared to the SN nodes, the high temporal flexibility nodes in the CON and FPN have significantly lower spatiotemporal diversity, suggesting that these nodes have less spatially varied time-varying interactions with other communities than those in the SN. The highly flexible nodes in the FPN and subcortical regions had significantly lower network centrality for time-varying interactions when compared to those in the SN. Taken together, these results emphasize the unique dynamical functional properties of the SN.

***High temporal flexibility nodes***

In both Sessions 1 and 2, brain networks that contribute the majority of high temporal flexibility nodes were localized to the SN, CON, FPN and subcortical nodes. The high temporal flexibility nodes in the SN were located in the bilateral anterior insula, dorsal anterior cingulate cortex, and frontal pole; those in the CON were also located in the bilateral insula and anterior cingulate cortex. Nodes with high temporal flexibility in the FPN were located at the bilateral frontal pole, middle frontal gyrus, precentral gyrus, right superior parietal lobule and supramarginal gyrus, and those in subcortical regions were located in the bilateral putamen and thalamus. Interestingly, the high temporal flexibility nodes located in the insula and cingulate cortex from the SN and CON are anatomically proximal. Importantly, while such an overlap reflects limitations of community labels assigned on the basis of previous static network analysis [[1](#_ENREF_1), [2](#_ENREF_2)], multiple nodes of bilateral anterior insula and dorsal anterior cingulate cortex were consistently identified as highly temporal flexible nodes.

***High temporal flexibility of subcortical nodes***

A surprising finding of our study is that subcortical nodes also showed a high level of temporal flexibility. However, unlike the SN, temporal flexibility of subcortical nodes did not predict cognitive flexibility (*ps* > 0.05 in both Session 1 and 2 data). Because nodes of the subcortical “network” from the Power et al atlas [[1](#_ENREF_1), [2](#_ENREF_2)] do not provide adequate sampling of the subcortical nodes and, furthermore, do not distinguish between the basal ganglia and the thalamus on the one hand, and the ventral and dorsal striatum on the other hand, the precise functional distinctions were not explored in the current study. Our findings are, however, consistent with the large body of animal research demonstrating the critical role of the basal ganglia and thalamus in dynamic control of cortical circuits [[3](#_ENREF_3), [4](#_ENREF_4)]. In humans, the caudate has been shown to dynamically link signals in distinct functional networks during different phases of a Sternberg working memory task [[5](#_ENREF_5)]. The extent to which the temporal flexibility of subcortical nodes, as identified in the present study, contributes to integrative and adaptive cognitive and motor functions of the basal ganglia and thalamus remains an important topic for further research.

***Temporal dynamics on a shorter time-scale***

To investigate whether the unique features associated with SN dynamics are also observed on a shorter time-scale, we repeated our analysis using a shorter window of 20 seconds. Results of this analysis (**S2** **Fig**) were identical to those reported in the original manuscript which were obtained with a window length of 40 seconds. Similar to the main analysis, the temporal flexibility of SN was significantly higher than the FPN. Importantly, we obtained the same results in Session 1 and Session 2 data. It should be noted that an exponentially tapered sliding window [[6](#_ENREF_6), [7](#_ENREF_7)] approach puts more weights on most recent observations and less on more distant observations, which helps to increase the sensitivity for capturing temporal dynamics using short windows [[6](#_ENREF_6), [7](#_ENREF_7)].

***Relation between temporal flexibility and static time-averaged network measures***

We performed additional analysis examining the relation between temporal flexibility and two static network measures: the static participation coefficient and the node strength. Participation coefficient is a measure of the diversity of inter-community connections of individual nodes, and node strength measures the total connection strengths of each node with all the nodes in the network. Participation coefficient and node strength were computed for positive static time-averaged functional connections using the Brain Connectivity Toolbox [[8](#_ENREF_8)].

We found that the relation between temporal flexibility and participation coefficient was not linear. For nodes with low temporal flexibility, participation coefficient was positively correlated with temporal flexibility (*r* = 0.70*, p* < 0.001; **S3** **Fig panel A**). In contrast, for nodes with high temporal flexibility, participation coefficient was negatively correlated with temporal flexibility (*r* = -0.47, *p* < 0.001; **S3** **Fig panel A**). Similar results were also observed in the Session 2 data (*r* = 0.68, *p* < 0.001 for low temporal flexibility nodes, **S3 Fig panel C**; *r* = -0.27, *p* < 0.001 for high temporal flexibility nodes, **S3** **Fig panel C**). These results demonstrate that our temporal flexibility measures capture connectivity features different from what might be predicted by static participation coefficient measures.

We then repeated the above analyses using static node strength and found similar results. The relation between temporal flexibility and node strength was highly nonlinear. For nodes with low temporal flexibility, static node strength was negatively correlated with temporal flexibility (*r* = -0.42*, p* < 0.001; **S3 Fig panel B**). In contrast, nodes with higher temporal flexibility did not show a significant correlation between static node strength and temporal flexibility (*r* = 0.03, *p* = 0.34; **S3 Fig panel B**). Similar results were also observed in the Session 2 data (*r* = -0.39, *p* < 0.001 for low temporal flexibility nodes, **S3 Fig panel D**; *r* = 0.01, *p* = 0.87 for high temporal flexibility nodes, **S3 Fig panel D**). These results further demonstrate that temporal flexibility captures connectivity features different from what might be predicted by static node strength measures.

***Static time-averaged network measures do not predict cognitive flexibility***

To further demonstrate the specificity of SN temporal flexibility measures in predicting behavior, we examined two static SN measures: participation coefficient and node strength. Canonical correlation analysis with cross-validation and prediction analysis procedures were used to predict cognitive flexibility. In contrast to SN temporal flexibility, we found no significant relationship between cognitive flexibility and either static network measures (*ps* > 0.05).

***Comparison with noise model***

To compare our findings with a noise model and to examine the sensitivity of our metrics of dynamic temporal flexibility, we conducted several additional analyses. We first examined SN metrics relative to those of other networks in a “noise” model [[9](#_ENREF_9)]. The noise model was constructed using 100 surrogate datasets; each surrogate dataset was obtained by applying a Fourier transform to the observed signal in each region. We randomized the phase by adding a random phase shift sampled in the interval [0, 2π]. Shifts at the same frequency from different time series and at different frequencies were all chosen independently. An inverse Fourier transform was then applied to generate an instance of surrogate data. Randomization of the phase response creates a stationary stochastic time series with the same auto-correlation structure as the original data but without non-stationarities or cross correlations. We then repeated our entire analysis on the surrogate data generated by the noise model.

Results of this analysis revealed that, in contrast to the experimental data, the temporal flexibility of the SN in the noise model was not higher than any other network (*ps* > 0.3 for both Session 1 and 2 data). Furthermore, the temporal flexibility of the SN in the experimental data was significantly lower than those obtained by the noise model (*ps* ≤ 0.05 for both Session 1 and 2 data). We then examined the temporal flexibility across all nodes. We found that temporal flexibility was significantly lower (*p* < 0.01) in the experimental data, compared to the noise model. Taken together, these results confirm that our experimental findings do not arise from trivial fluctuations due to noise, finite sample effects or cross-correlations in the data.

**References**

1. Power JD, Cohen AL, Nelson SM, Wig GS, Barnes KA, Church JA, et al. Functional network organization of the human brain. Neuron. 2011;72(4):665-78. doi: 10.1016/j.neuron.2011.09.006. PubMed PMID: 22099467; PubMed Central PMCID: PMC3222858.

2. Power JD, Schlaggar BL, Lessov-Schlaggar CN, Petersen SE. Evidence for hubs in human functional brain networks. Neuron. 2013;79(4):798-813. doi: 10.1016/j.neuron.2013.07.035. PubMed PMID: 23972601; PubMed Central PMCID: PMC3838673.

3. Kravitz AV, Freeze BS, Parker PR, Kay K, Thwin MT, Deisseroth K, et al. Regulation of parkinsonian motor behaviours by optogenetic control of basal ganglia circuitry. Nature. 2010;466(7306):622-6. doi: 10.1038/nature09159. PubMed PMID: 20613723; PubMed Central PMCID: PMC3552484.

4. Liu J, Lee HJ, Weitz AJ, Fang Z, Lin P, Choy M, et al. Frequency-selective control of cortical and subcortical networks by central thalamus. eLife. 2015;4. doi: 10.7554/eLife.09215. PubMed PMID: 26652162; PubMed Central PMCID: PMC4721962.

5. Chang C, Crottaz-Herbette S, Menon V. Temporal dynamics of basal ganglia response and connectivity during verbal working memory. NeuroImage. 2007;34(3):1253-69. doi: 10.1016/j.neuroimage.2006.08.056. PubMed PMID: 17175179.

6. Pozzi F, Di Matteo T, Aste T. Exponential smoothing weighted correlations. Eur Phys J B. 2012;85(6):175.

7. Zalesky A, Fornito A, Cocchi L, Gollo LL, Breakspear M. Time-resolved resting-state brain networks. Proceedings of the National Academy of Sciences of the United States of America. 2014;111(28):10341-6. PubMed PMID: 24982140; PubMed Central PMCID: PMC4104861.

8. Rubinov M, Sporns O. Complex network measures of brain connectivity: uses and interpretations. NeuroImage. 2010;52(3):1059-69. doi: 10.1016/j.neuroimage.2009.10.003. PubMed PMID: 19819337.

9. Ryali S, Supekar K, Chen T, Menon V. Multivariate dynamical systems models for estimating causal interactions in fMRI. NeuroImage. 2011;54(2):807-23. doi: 10.1016/j.neuroimage.2010.09.052. PubMed PMID: 20884354; PubMed Central PMCID: PMC2997172.
